# Microfluidic Chip-Based Cancer Diagnosis and Prediction of Relapse by Detecting Circulating Tumor Cells and Circulating Cancer Stem Cells

**DOI:** 10.3390/cancers13061385

**Published:** 2021-03-18

**Authors:** Hyeon-Yeol Cho, Jin-Ha Choi, Joungpyo Lim, Sang-Nam Lee, Jeong-Woo Choi

**Affiliations:** 1 Department of Bio & Fermentation Convergence Technology, Kookmin University, Seoul 02707, Korea; chohy@kookmin.ac.kr; 2 Interdisciplinary Program for Bio-health Convergence, Kookmin University, Seoul 02707, Korea; 3 Department of Chemical and Biomolecular Engineering, Sogang University, Seoul 04107, Korea; jhchoi@jbnu.ac.kr (J.-H.C.); jpim92@sogang.ac.kr (J.L.); 4 School of Chemical Engineering, Jeonbuk National University, Jeonju 54896, Korea; 5 Uniance Gene Inc., 1107 Teilhard Hall, 35 Baekbeom-Ro, Mapo-Gu, Seoul 04107, Korea

**Keywords:** liquid biopsy, microfluidic platform, circulating tumor cells, circulating cancer stem cells, optical sensing, early diagnosis

## Abstract

**Simple Summary:**

Metastasis is the main cause of cancer-related death. Circulating cancer stem cells have recently attracted attention because they have higher tumorigenicity than non-stem-like circulating tumor cells. Despite the strong scientific evidence for circulating cancer stem cells and secondary tumor formation, the exact mechanisms behind the generation and characteristics of circulating cancer stem cells are not yet fully understood because of their extreme scarcity. This review aims to introduce the recent advances in the detection and analysis of circulating tumor cells and circulating cancer stem cells.

**Abstract:**

Detecting circulating tumor cells (CTCs) has been considered one of the best biomarkers in liquid biopsy for early diagnosis and prognosis monitoring in cancer. A major challenge of using CTCs is detecting extremely low-concentrated targets in the presence of high noise factors such as serum and hematopoietic cells. This review provides a selective overview of the recent progress in the design of microfluidic devices with optical sensing tools and their application in the detection and analysis of CTCs and their small malignant subset, circulating cancer stem cells (CCSCs). Moreover, discussion of novel strategies to analyze the differentiation of circulating cancer stem cells will contribute to an understanding of metastatic cancer, which can help clinicians to make a better assessment. We believe that the topic discussed in this review can provide brief guideline for the development of microfluidic-based optical biosensors in cancer prognosis monitoring and clinical applications.

## 1. Introduction

Over the past few decades, the development of anticancer drugs has successfully advanced through an ever more systematic understanding of cancer biology afforded by emerging research insights into the details of cancer genomics, metabolism, microenvironment, and other factors that affect the outcomes of the disease [1,2]. While the expected life span of cancer patients has been extended with new therapeutic strategies, cancer has remained in the upper ranks when it comes to overall mortality from diseases [3]. Over 90 percent of cancer deaths are due to distant metastasis from the primary tumor site [4,5]. Therefore, it is essential to develop a novel strategy that can identify the presence of metastatic cancer early and analyze its characteristics. To this end, an analysis method using “liquid biopsy” has emerged.

As cancer progresses, information about cancer circulates in various forms in the blood and in various tissues throughout the body, at the DNA, protein, exosome, and cellular levels [6]. This information can be collected in a blood sample and analyzed to characterize cancer, a method referred to as “liquid biopsy” [7]. Compared to existing biopsy methods, liquid biopsy offers advantages in monitoring metastatic cancer because it not only minimizes the pain burden of the patient but also allows repeated sample collection. However, this method has only recently been used in clinical practice [8]. A major challenge of using circulating cancer biomarkers is detecting extremely low concentrations of targets amidst the high noise introduced by the presence of serum and hematopoietic cells [9]. Various methods have been developed to overcome these limitations; among them, the microfluidic technique is considered the most suitable method for selective isolation, enrichment, and target-specific analysis of circulating cancer biomarkers [10].

Here, our group applies its combined expertise in bioengineering and chemistry to provide a comprehensive summary of the contributions of microfluidics and optical sensing methods, two major detection techniques for circulating tumor cells (CTCs) and circulating cancer stem cells (CCSCs) specifically, to liquid biopsy technology and applications.

## 2. Circulating Tumor Cells (CTCs) and Circulating Cancer Stem Cells (CCSCs)

### 2.1. Circulating Tumor Cells (CTCs)

CTCs are floating tumor cells that traverse the body through the blood vessels and lymph nodes from the primary tumor or its metastases into the bloodstream, and they have been distinguished as epithelial cancer cells [7,11,12,13,14]. There is increasing interest in clinical applications for liquid biopsy, particularly with respect to CTCs, such as identifying biomarkers for the early detection of cancers, prediction of prognosis and metastasis, and monitoring of drug efficacy against cancers [7,8,15,16]. CTCs are closely related to distant metastases and are utilized as biomarkers of minimal residual disease (MRD) [17,18,19]. Biomarkers have been established for diagnosing and monitoring metastatic cancers and drug response in patients based on cancer type or based on the presence and quantity of tumor-specific markers on the cancer cells. Analysis of CTCs provides an easily repeated and minimally invasive method to regularly monitor changes in tumor cells that have the potential to launch and proliferate on new metastatic sites. Therefore, CTCs have been utilized as precise predictive and prognostic material in patients to examine localized, circulating, metastatic, and recurring diseases. However, the tiny traces and heterogeneity of CTCs in body fluids such as blood, urine, and saliva place significant limitations on the isolation and detection of CTCs. For example, several studies have found fewer than ten CTCs, compared with nearly 10 million white blood cells (WBCs) and 5 billion red blood cells (RBCs), in 1 mL of whole blood [20,21,22]. To overcome this limitation, diverse sensitive analytical methods with appropriate separation strategies into microfluidic devices have been developed. The separate problem of the heterogeneity of CTCs is reflected in differences in protein expression on their surface membranes and variability in the ratios of cellular contents, such as mRNA, miRNA, and other small molecules, depending upon which specific CTC is released from any given tumor [23,24,25]. Thus, the isolation and analysis of various CTCs can provide detailed and specific information about tumor type, progression, metastasis, and response to drug treatment. In addition, this characterization of CTCs holds promise for guiding personalized therapies and discovering novel drugs with better medicinal effects, especially when targeting the metastatic process.

### 2.2. Circulating Cancer Stem Cells (CCSCs)

CCSCs are subpopulations of CTCs which express stem cell markers, including CD24, CD44, CD133, CD166, and aldehyde dehydrogenase isoform 1 (ALDH1) [26,27,28]. CCSCs, which have the capability of the generation of new metastatic tumors, usually occur at a rate of less than 5% of CTCs. Due to this capacity for generating new metastases, they are referred to as tumor-initiating cells (TICs). Tumors are composed largely of non-tumorigenic cells and a few tumorigenic cells, CCSCs, or TICs. The relatively rare CTCs represent a minute percentage of the total blood cells in circulation, ranging from only 1 to 100 CTCs/mL of blood, among 4 × 10^9^ blood cells. CCSCs are expected to be present in circulation at a proportion of 0.01–2% of bulk CTCs [29,30]. Therefore, the total occurrence of CCSCs is extremely rare. They can self-renew, extensively proliferate, and generate differentiated descendants, similarly to typical stem cells [31]. These cells show distinct tumorigenic activity in xenograft transplantation models such as immunodeficient mice, which verifies their crucial role in cancerization [32]. Therefore, CSCs are regarded as the root of a tumor. The CCSC is typically resistant to diverse cancer treatments such as chemotherapy, hypoxia, and radiotherapy [33]. Reliable identification of CCSCs is thus necessary, and therapies targeted to CCSCs have considerable potential in the management of metastatic cancer. However, the detection CCSCs is limited by a lack of clear understanding of their molecular characteristics, such as the precise surface markers that identify subsets of CCSCs according to their aggressiveness or drug susceptibility. Precise measurement of CCSCs is also technically challenging, due to their rare occurrence in the CTC population relative to a much larger background of blood cells. Therefore, an entirely novel in vitro diagnostic platform is required to detect these extremely low concentrations of CCSCs.

## 3. Enrichment of Cancer-Related Circulating Cells

### 3.1. Nanoparticle-Assisted Enrichment Strategy

**Figure 1:**
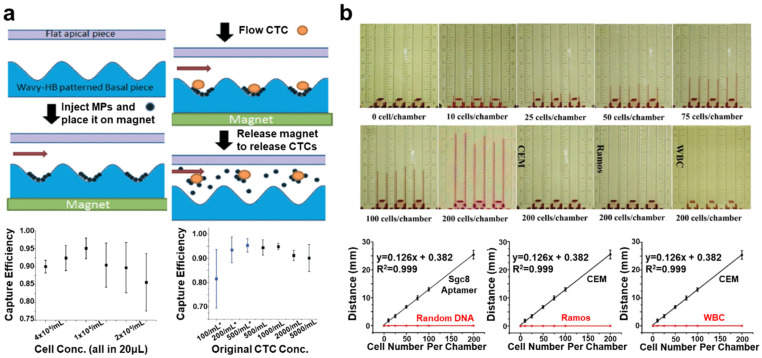
(**a**) Schematic images of capture, the release of circulating tumor cells (CTCs), and capture efficiency of CTCs using wavy-herringbone-structured microfluidic devices. This figure is reproduced from [40] (© 2017 The Royal Society of Chemistry); (**b**) Quantitative detection of CTCs using volumetric bar-chart chip by analysis of distance moved by ink, proportional to CTC concentration. This figure is reproduced from [41] (© 2019 John Wiley & Sons).

Isolation and enrichment of CTCs and CCSCs from body fluids in liquid biopsies, with minimally invasive methods, hold prodigious potential for early cancer diagnosis and evaluation of therapeutic efficacy. To date, diverse platforms have been developed to efficiently separate CTCs and CCSCs. Among them, immunomagnetic separation by specific antibody-functionalized magnetic nanoparticles is the most frequently used strategy [34]. This technique offers several advantages, owing to the fast magnetic response, high surface area, and good biocompatibility of magnetic nanoparticles. Nie et al. developed folic acid (FA)-functionalized magnetic iron oxide nanoparticles for direct ovarian cancer CTC separation from the blood of clinical ovarian cancer patients [35]. In this system, a two-step binding mechanism was used to increase the number of nanoparticles attached to the CTCs, thereby improving their capture efficiency. FA-functionalized magnetic nanoparticles (MNPs) and streptavidin-MNPs were attached to the surfaces of CTCs simultaneously, using biotin–bovine serum albumin (BSA)–FA. Using this two-step binding system, CTCs were successfully separated from patients’ blood samples with significant isolation efficiency in the absence of prior pretreatment. Chang et al. further showed that two fluorescent magnetic mesoporous silica nanoparticles (M-MSNs) with rod- and sphere-shaped forms could be used to isolate CTCs [36]. To attach to the CTCs, the anti-epithelial cell adhesion molecule (anti-EpCAM) antibody was functionalized on the different shapes of M-MSNs. Rod-shaped M-MSNs exhibited faster enrichment of CTCs in spiked cells and real samples than the sphere-shaped M-MSNs. These results verified that the shape of M-MSNs could affect their interaction with CTCs and their separation efficiency. Meng et al. also developed RBC membrane-coated magnetic nanoparticles with the anti-EpCAM modification [37]. RBC membrane-coated particles were prevented from adsorbing non-specific biomolecular interactions in protein-enriched plasma, such as blood. Using spiked blood samples, they found that the isolation of PC-3 cells using RBC-magnetic nanoparticles was superior to the non-functionalized magnetic nanoparticle, increasing efficiency from 60.22% to 95.71%. Wu et al. demonstrated the superparamagnetic positively charged nanoparticle (SPPCN)-based isolation of CTCs from the real blood samples of 25 colorectal cancer patients [38]. Due to the negative surface charge of CTCs, serum protein-coated, positively charged magnetic nanoparticles can trap different types of CTCs according to their surface protein expression. In this study, CTCs were separated and identified in 1 mL of blood samples from all 25 colorectal cancer patients. For the isolation of CTCs, nanoparticle-integrated microfluidic devices have been employed to maximize efficiency by integrating magnetic force. Zhao et al. presented a laminar-flow microfluidic ferrohydrodynamic cell separation (FCS) device which was able to enrich rare CTCs. It could separate the CTCs with high throughput (6 mL h^−1^), high purity of low concentrations (11.7% purity in ~100 cells mL^−1^), and a high rate of recovery (92.9%), in a biocompatible manner [39]. This microfluidic system took advantage of the magnetic buoyancy force to sort magnetic nanoparticle-functionalized CTCs according to their size while maintaining their viability and the surface expression of specific proteins. Shi et al. used a wavy-herringbone-structured microfluidic device to separate rare CTCs using anti-EpCAM functionalized magnetic nanoparticles (Figure 1a) [40]. CTC-capturing magnetic nanoparticles were trapped over the periodic U-shaped site in the wavy-herringbone on the polydimethylsiloxane (PDMS) surface by an external magnetic field and were released by removing the magnetic force. The capture efficiency from whole blood averaged 81.5 ± 12.0% in a low concentration, as low as 100 mL^−1^ of the HCT-116 cells. Abate et al. developed a simple and portable microfluidic device that enabled CTC collection with a highly sensitive (single-cell resolution) visual quantitative detection module (Figure 1b) [41]. An aptamer-functionalized magnetic nanoparticle was tagged onto the CTCs and separated by magnetic force. After sorting, CTCs were detected in a volumetric bar-chart chip by colorimetry, using a platinum nanoparticle, hydrogen peroxide, and ink. The authors claimed that this microfluidic platform was sensitive enough to quantify the CTCs, even at the level of a single CTC cell, by a change in distance moved by the ink.

In the aforementioned studies, the immunomagnetic nanoparticle is utilized to label the epithelial marker expressed cells for the isolation of CTCs. On the other hand, it also has been used to eliminate the potential contaminants, WBCs, from the blood sample. Even though this requires additional steps, the commercialized CTC enumeration platforms, such as CellSearch and IsoFlux, involve both positive and negative selection processes with magnetic nanoparticles to improve the detection reliability of sorted cells.

### 3.2. Direct Capturing on the Microfluidic Device with Nano- and Microstructures

**Figure 2:**
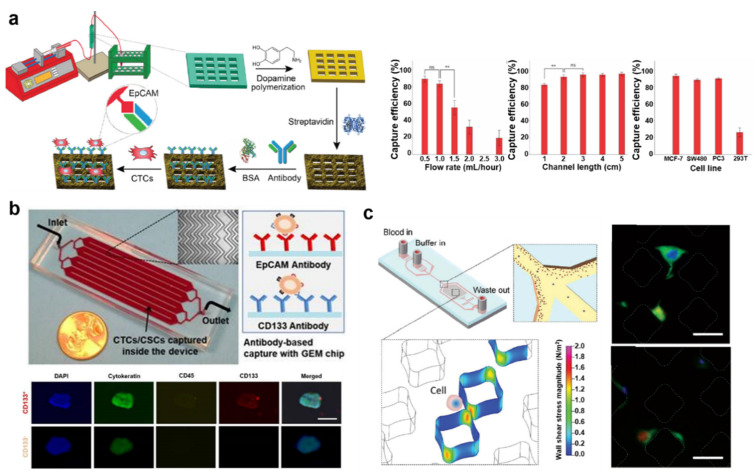
(**a**) Schematic images of the fabrication process of 3D-printed microfluidic device for CTC isolation; capture efficiency of CTCs under different conditions is quantified. This figure is reproduced from [45] (© 2019 Elsevier B.V.); (**b**) Schematic image of the geometrically enhanced mixing microfluidic chip for CTC and circulating cancer stem cell (CCSC) counting. This figure is reproduced from [46] (© 2019 Ivyspring International Publisher); (**c**) Schematic images of size-based CTC separation using microfluidic chip and growth of separated CTCs from blood on the microfluidic chip. This figure is reproduced from [50] (© 2020 The Royal Society of Chemistry).

Besides magnetic nanoparticle-assisted isolation of cells for liquid biopsy, a direct capturing strategy was also developed in a microfluidic device. In this analytical method, biomolecules such as antibodies and aptamers conjugated to the surface proteins of CTCs and CCSCs are immobilized on a specific section of the microfluidic device. Kim et al. developed a graphene oxide (GO)-functionalized microfluidic device for the isolation of CTCs with particular channel geometry for uniform flow distribution [42]. Control of flow distribution improved the isolation purity of CTCs, and multiple analyses were possible in one microfluidic device. In this device, GO played an important role in widening the surface area for the separation of CTCs. Using this platform, metastatic breast cancer (MBC) patient-derived CTCs were successfully isolated from a one-milliliter blood sample. Isolated exosomes were analyzed by immunofluorescence methods and qRT-PCR was used for CTC expression analysis. This strategy revealed interpatient heterogeneity of oncogenic signatures, such as epithelial-to-mesenchymal transition (EMT) and apoptotic-resistant mechanisms. Zeinali et al. described a system of two-connected CTC carpet chips, composed of micro-sized posts, with antibody functionalization [43]. This dual capture strategy using anti-EpCAM and anti-CD133 facilitated the isolation of distinct, heterogeneous CTC populations (epithelial CTCs and EMT cells) simultaneously from pancreatic cancer patient samples with over 97% recovery and 76% purity. Collected CTCs using this sequential microfluidic device could be further analyzed for specific gene expression related to metastasis and prognosis. The authors claimed that targeting genes integral to the EMT process and personalized therapy could reduce metastasis and increase the survival rate of cancer patients. Loeian et al. produced a nanotube-CTC-chip, which consisted of film-typed carbon nanotubes and electrodes, with microarray batch manufacturing techniques [44]. This 76-element microarray was used to enrich CTCs based on the excellent adherent property of the carbon nanotube. Compared to collagen adhesion matrix (CAM) scaffolding, carbon nanotube scaffolding showed over 90% adherence and 100% tracking efficiency. Remarkably, this device could be used to identify single CTCs exhibiting multiple phenotypes in the early (CK8/18, EGFR) and advanced stages (Her2, EGFR) of breast cancer. Chen et al. developed a 3D-printed microfluidic device with anti-EpCAM functionalization for the isolation of CTCs from the bodily fluid (Figure 2a) [45]. The 3D printing strategy increased the surface area drastically by permitting manipulated fluid flow patterns. In this manner, 3D-printed objects were integrated into the microfluidic device layer-by-layer, and anti-EpCAM immobilization was employed. This process yielded capture efficiencies of the CTCs from different cancer cells of up to 92.42 ± 2.00% (MCF-7), 87.74 ± 1.22% (SW480), and 89.35 ± 1.21% (PC3). Similarly, Varillas et al. immobilized antibodies against a CSC biomarker, CD133, on the surface of a microfluidic platform for the treatment monitoring of patients by tracking the CTCs and CCSCs in the patient’s blood (Figure 2b) [46]. The microfluidic channel was designed with a herringbone structure to enhance the mixing of the microfluidic fluid. This method revealed that the majority (84.4%) of patient blood samples were positive for CTCs, and 70.8% of samples were positive for CSCs. Further, their numbers decreased with clinical treatment.

### 3.3. Density-Based Isolation on the Microfluidic Device

Due to the mass and density of the CTCs and CCSCs in body fluid, they can be separated or discriminated from the residual biomaterials using dynamic fluidic flow in microfluidic devices and analyzed for cancer-related information. Several attempts at CTC isolation have been made by manipulating microfluidic flow. Chiu et al. developed a cell manipulating microfluidic system integrated with optically induced dielectrophoresis (ODEP) for the isolation of CTCs according to size [47]. ODEP-based techniques offer a simple, non-contact method of cell manipulation. ODEP generates a non-uniform electric field by light illumination, which can then be used to manipulate the electrically polarized CTCs. Using this device, it is possible to isolate integral CTC clusters with high cell purity (91.5 ± 5.6%), at a high recovery rate (70.5 ± 5.2%). Antfolk et al. presented a novel integrated microfluidic device that enabled acoustofluidic label-free isolation, direct dielectrophoretic trapping, and observation of single live cells [48]. The combined microfluidic system executed the separation, concentration, and trapping of single live CTCs for automated analysis without sample transfer. This method reduced the analysis time of the isolated and trapped CTCs and did not require labeling methods such as antibodies or other affinity-based molecules. Xue et al. demonstrated the continuous-flow separation of CTCs in the dynamic Halbach array magnet-integrated microfluidic device [49]. The dynamic movement of the Halbach array magnet created a continuous magnetic force toward the inside of the microfluidic channel and induced movement of magnetic bead-labeled WBCs, thereby segregating them from the CTCs. This device efficiently captured CTCs from whole blood with high throughput (6 mL h^−1^) and yielded a high average capture rate of more than 90.0% at an optimal condition (flow rate, 100 mL min^−1^; concentration of CD45-labeled immunomagnetic beads, a ratio of 20:1). Zhou et al. reported a simple microfluidic chip system, enabling the on-chip separation, capture, and immunofluorescence assay of CTCs simultaneously (Figure 2c) [50]. This microfluidic device consisted of an upstream channel for cell isolation by size and a downstream chamber for cell trapping by a micropost array and culture system. Isolated cells could easily be cultured by adding a growth medium at the inlet ports every 12 h, which was possible without a pumping system. The authors successfully separated Hep G2 cells from blood samples and cultured them in situ on-chip for more than 10 days without the need for a bulky pumping system and obtained a 68% survival rate.

In summary, for CTC isolation, several biological materials, such as RBCs and WBCs, in the biological complex could interrupt and decrease the isolation efficiency. Therefore, it is essential to show the CTC isolation efficiency using a real sample. In order to verify the efficient isolation of CTCs in each of the studies, a whole-blood sample or WBC-mixed buffer solution was used in place of the real patient sample (Table 1). In most of the isolation studies using specific biological binding, isolation efficiency was exhibited above 90%. On the other hand, the label-free method exhibited low isolation efficiency from a spiked human blood sample. These results showed that specific biological interactions such as immune separation or aptamer-based isolation are better than density-based isolation in terms of isolation efficiency. Moreover, the change in the surface marker expression profiles also affects the CTC isolation efficiency [51]. The epithelial–mesenchymal transition (EMT) process induces a decrease in the epithelial marker expression and has the potential to generate hybrid phenotypes [52]. After EMT induction in breast cancer cells (MCF-7), EpCAM was downregulated by more than 50% [53]. This result indicates that the CTC detection efficiency can be decreased when the platform uses a single antibody-based detection platform, such as CellSearch. To overcome this potential problem, we introduce various multi-target-based optical analysis platforms for CTC detection in the next section.

## 4. Optical Analysis Platform of CTCs and CCSCs for Phenotyping Primary Cancer

### 4.1. Fluorescence-Based Optical Detection of CTCs and CCSCs

After the sensitive and effective isolation and enrichment of CTCs and CCSCs, identifying phenotypic characteristics of primary cancer is an essential process for patient-specific treatment and understanding of cancer behavior. Considering the distinct limitation of sample isolation and analysis of CTCs, microfluidic platforms have been widely used in combination with a fluorescence detection system for multiplexing analysis. As mentioned in Section 3.2 above, capturing biomolecules anchors the CTCs and CCSCs directly to a specific section of the microfluidic device. To analyze these captured CTCs and CCSCs, fluorescent-dye-labeled antibodies are applied and washed through the microfluidic channel. To identify the CTCs and CCSCs, a broad spectrum of cancer-related cell surface markers has been used (Table 2) [54,55,56,57,58,59,60,61,62,63,64,65,66,67,68,69,70,71,72,73].

**Figure 3:**
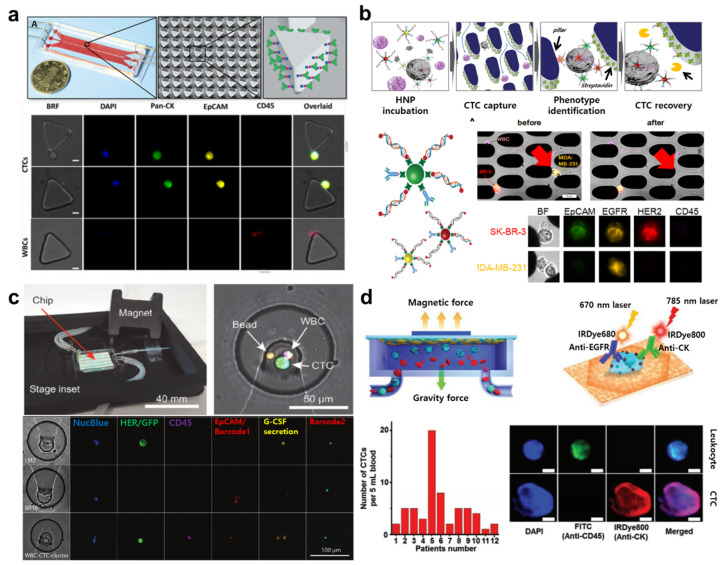
(**a**) Schematic images of size-dictated immunocapture chip (SDI-Chip) and analysis of surface marker of captured CTCs. This figure is reproduced from [74] (© 2017 John Wiley & Sons); (**b**) Schematic images of hybrid nanoparticle-based CTC capture using microfluidic chip and selective recovery of captured CTCs from the microfluidic chip. This figure is reproduced from [64] (© 2013 Elsevier B.V.); (**c**) Microfluidic chip design and operation for CTC capture and protein secretion analysis. This figure is reproduced from [75] (© 2020 John Wiley & Sons); (**d**) Schematic images of a plasmonic gold chip used in the microfluidic immunomagnetic method and screening of CTCs in cancer patients. This figure is reproduced from [76] (© 2018 John Wiley & Sons).

After the first report by Nagrath et al. of microfluidic device-based CTC detection by immobilizing anti-EpCAM antibodies on micropillars in the microfluidic channel, many similar approaches have been published [54]. Ahmed et al. developed a size-dictated immunocapture chip with a triangular microarray structure that can selectively enhance the interaction of CTC by deterministic lateral displacement (Figure 3a) [74]. The anti-EpCAM antibody-coated micropillars successfully captured more than 90% of CTCs (92.2 ± 6.4%). Even though WBCs were well distinguished from CTCs by multiplex immunofluorescence staining on the chip, staining requires several steps for each antibody, including washes, and captured CTCs may be lost at each of these steps. To overcome this issue, Lee et al. introduced a hybrid fluorescence nanoparticle-based CTC capture and analysis system (Figure 3b) [64]. The hybrid fluorescence nanoparticle (HNP) is composed of a quantum dot, antibody, and biotinylated DNA, which constitute a signaling element, CTC labeling element, and capturing element, respectively. Streptavidin was used to coat the micropillars in a microfluidic channel to hold the biotinylated DNA of HNPs, which resulted in the successful capture of the target CTCs. This methodology allowed visual discrimination of the captured CTCs, which were labeled in different colors depending on surface marker expression due to the ratio of different HNPs which individually recognized the antibody and quantum dots. Moreover, the DNA of HNP served as a cleavable linker, whereby a restriction enzyme was used to recover captured CTCs by mild cleavage of DNA. Such recovery of CTCs from the microfluidic device enables further analysis of the captured CTCs, which in turn better informs clinical decisions. Recently, Armbrecht et al. introduced another tool to analyze cytokine secretion from captured CTCs within a microfluidic system (Figure 3c) [75]. Using this system, the secretion level of granulocyte colony-stimulating factor (G-CSF), which indicates acute inflammation, was directly quantified. These advanced microfluidic sensing systems have granted researchers access to direct proteomic profiling of CTCs, permitting a better understanding of the molecular pathways and signals involved in the metastatic process.

The intensity of the fluorescence signal of immune-labeled CTCs and CCSCs correlates directly with surfacer marker expression. To enhance the fluorescence signal of captured CTCs, Zhang et al. introduced a magnetic “squashing” technique on the plasmonic gold (pGOLD) chip in a microfluidic device (Figure 3d) [76]. After CTCs were magnetically captured on the pGOLD chip, near-infrared (NIR) fluorescence enhancement (≈50–120-fold) was used to interrogate the squashed/flattened morphology of CTCs by magnetic forces. Due to the proximity of NIR labels on CTCs to the plasmonic gold chip, the fluorescence signal was enhanced by surface plasmon resonance. This research holds potential for CCSC detection. Interestingly, CCSCs showed greater cytoskeletal and nucleoskeletal deformability and motility compared to CTCs [77]. By monitoring their deformation and motion within the microfluidic device, Zhang et al. were able to distinguish the more highly tumorigenic cells, CCSCs, from surrounding CTCs. Although the effect of squashing on cell viability was not described in this study, mechanical modulation of the cell was successfully executed for fluorescence detection of circulating cells.

### 4.2. Raman Spectroscopy-Based Optical Detection of CTCs and CCSCs

Although fluorescence and surface plasmon resonance (SPR) detection methods are the most well-established optical sensing tools for a microfluidic device, there is still a need to improve the multiplexity of these methods. The wide variety of required sets of antibodies for each primary organ is one challenge associated with the identification of CTCs using surface marker expression analysis. To this end, surface-enhanced Raman spectroscopy (SERS) has been introduced as a new tool for optical sensing platforms. Raman spectroscopy has significant advantages over fluorescence imaging, including minimizing the background noise from the blood that results from autofluorescence signals. Distinctive non-overlapping peaks are detected from a large pool of chemical dyes for multiplex imaging, allowing for more precise signal characterization.

**Figure 4:**
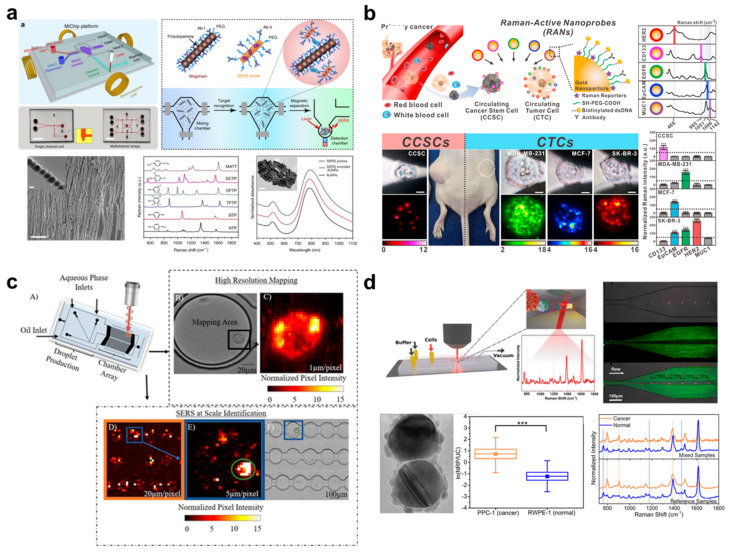
(**a**) Schematic illustration of the Magchain integrated microfluidic chip. This figure is reproduced from [78] (© BY-NC 4.0, 2018); (**b**) Illustration of Raman-active nanoprobe-based circulating cancer stem cell analysis. This figure is reproduced from [70] (© 2018 Elsevier B.V.); (**c**) Illustration of a single-cell encapsulation event within the microfluidic device. This figure is reproduced from [79] (© 2018 American Chemical Society); (**d**) Graphical depiction of device layout and flow dynamics. This figure is reproduced from [80] (© 2015 American Chemical Society).

To sense CTCs and CCSCs with SERS, metal nanoparticles are typically used, as they offer specific advantages for isolating and enriching targets found in the blood. However, the high mobility of CTCs and CCSCs makes it difficult to find the focal point of the laser on the SERS-tagged cells. To overcome this technical limitation, magnetic nanoparticles have become a popular element in SERS probe design. To control the binding of cells with SERS probes magnetically in a microfluidic channel, Xiong et al. developed magnetic nanochains (Magchains) (Figure 4a) [78]. Similar to Zong’s approach, an antibody-conjugated Magchain and a gold nanorod-based SERS probe are mixed in the mixing chamber of the device and form sandwich immune complexes when the target is present. Sandwich complexes are then guided into the small-sized chamber in a microfluidic device by magnetic force. The use of such gathered complexes successfully enhanced the SERS signal of cancer biomarkers in this study. In an alternative approach, Cho et al. designed a new class of SERS probe comprising five families distinguished by unique sets of antibodies, Raman dye, and a double-stranded DNA linker (Figure 4b) [70]. One of these SERS probes was conjugated with an anti-CD133 antibody for isolating circulating cancer stem cells (CCSCs). Here, CCSCs were successfully isolated from a mixed population of CTCs and hematopoietic stem cells on the microfluidic device. Mapping results clearly showed distinctive signal differences according to surface marker expression. Willner et al. developed SERS droplet microfluidics for single-cell analysis of CTCs (Figure 4c) [79]. A single prostate cancer cell was trapped with SERS nanoprobes in the microfluidic device and isolated droplets were kept stationary during SERS interrogation. One drawback of this method is that mapping the SERS signal over a large area within the microfluidic device is a time-consuming process and negatively affects cell viability. Pallaoro et al. developed an integrated microfluidic SERS system that can identify and count cancer cells from a mixed population of cells flowing through a microfluidic channel (Figure 4d) [80]. In their study, CTCs were labeled with silver nanoparticle dimers conjugated with a Raman-active reporter molecule and passed through a flow-focused microfluidic channel, which forces the cells into a single line. Each cancer cell was correctly identified among a proportionally larger number of normal cells by their Raman spectra.

### 4.3. Colorimetry-Based Optical Detection of Circulating Cancer Biomarkers

**Figure 5:**
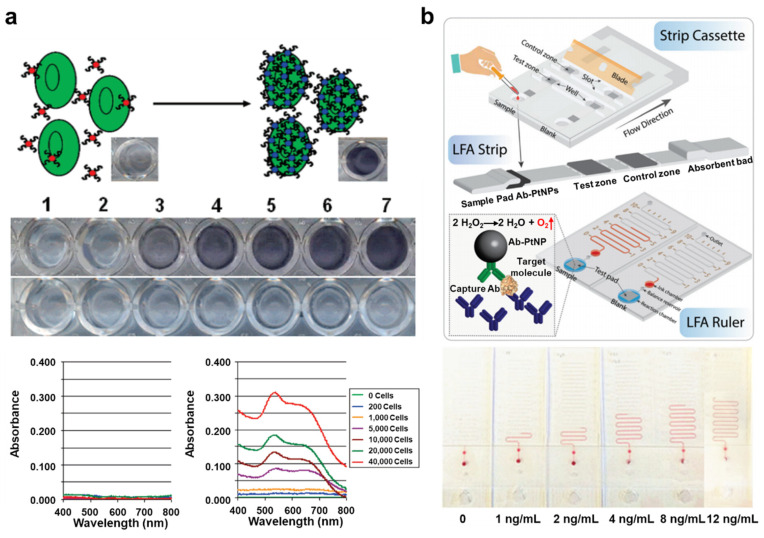
(**a**) Schematic images of aptamer-conjugated gold nanoparticle-based assay system and colorimetric analysis of target cancer cells. This figure is reproduced from [82] (© 2018 American Chemical Society); (**b**) Schematic images of lateral flow assay-based microfluidic device and verification process of the target molecule by distance traveled by ink. This figure is reproduced from [85] (© 2019 Royal Society of Chemical).

Similarly, many other studies have taken advantage of nanoparticles to detect CTCs (Figure 5a) [81,82]. The simplest and fastest of these methods is to conjugate the particles to nucleic acids, such as DNA and aptamer, that bind selectively with the overexpressed proteins on CTC membranes or nucleic acids in the target CTCs. As the nucleic acids selectively bind to the CTCs, the nanoparticles aggregate, forming a larger structure and inducing a color change in the CTC-containing solution. The higher the concentration of CTCs, the more nanoparticles are aggregated by the nucleic acids that selectively bind to the CTCs, resulting in color changes that vary according to the resulting change in absorbance. Lu et al. reported a multifunctional, oval-shaped, gold nanoparticle-based, selective breast cancer cell detection system [83]. In this study, the surfaces of oval-shaped gold nanoparticles were modified with an S6 RNA aptamer and an anti-HER2/c-erb-2 antibody to achieve high selectivity and sensitivity for a target cancer cell. This strategy made it possible not only to optically confirm the number of cancer cells in solution by the naked eye, but also to resolve the signal using a two-photon scattering assay in solutions with low concentrations of cancer cells. For more efficient discrimination of target cells among the various types of cells typically present in biological samples, Liu et al. developed microfluidic channels that permit aptamer-specific capture of target cells [84]. Target cells were captured inside of the microfluidic channel, then subjected to a flow of gold nanoparticle-conjugated aptamer. This microfluidic channel configuration detected target cells in short timescales and with relatively large volumes of samples. Li et el. introduced microfluidic devices that employ a lateral flow assay for quantitative and rapid point-of-caring tests (Figure 5b) [85]. This device used antibody-conjugated platinum nanoparticles to capture prostate-specific antigens. Platinum nanoparticle-catalyzed oxygen generated by H_2_O_2_ solution forces ink through the microfluidic device. This distance-based readout system provides rapid quantitation, eliminating the need for complex analytical equipment.

## 5. Outlook

In this review, we summarized recent strategies of isolation and analysis platforms for CTCs and CCSCs. Since CTCs need to be detected from blood samples in the presence of a tremendous number of RBCs and WBCs, the microfluidic platform is the most suitable system. Moreover, multi-probe-based optical analysis platforms are required not only to identify the origin and subtype of primary cancer but also to improve the detection reliability from heterogeneous phenotypes. Even though recent advances in optical analysis-based microfluidic devices have shown great success with CTC enumeration, they cannot quickly provide information about the entire sample because they can analyze only a fraction of the injected sample at a time. Thus, these optical analysis-based microfluidics approaches to analyzing various kinds of cancer biomarkers are low-throughput, despite their high sensitivity. Therefore, a more effective method for analyzing separated CTCs on microfluidic devices is needed to achieve clinical relevance.

**Figure 6:**
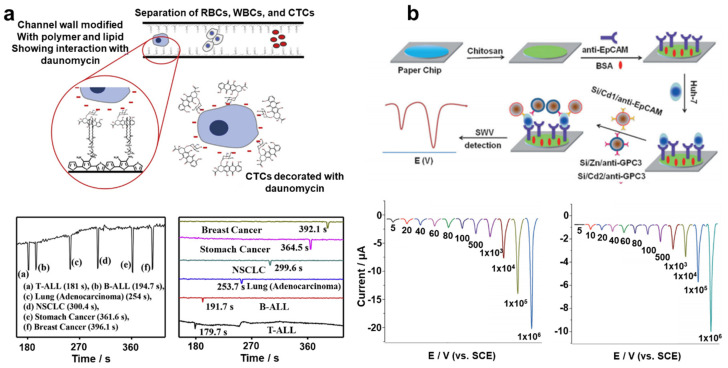
(**a**) Schematic images detailing the process of CTC capture using a functional channel wall and separation of target CTCs in mixed cancer cell samples. This figure is reproduced from [93] (© 2019 Elsevier B.V.); (**b**) Schematic image showing the fabrication and capture process of a paper-based microfluidic immunodevice and electrochemical signals obtained from CTCs detected in various concentrations. This figure is reproduced from [94] (© 2014 John Wiley & Sons).

For this reason, a variety of analytical processes were introduced into microfluidic devices, including electrochemical, fluorescence, and chemiluminescence techniques [64,86]. Among these methodologies, the electrochemical technique, which measures the electrical signal generated by the varying distribution of electrons that results from chemical reactions, has been widely adopted due to its uniquely high sensitivity, selectivity, and throughput [87,88]. Gurudatt et al. developed a microfluidic device for CTC separation by size variation and electrochemical distinction of their origin (Figure 6a) [72]. With this system, cancer patients’ samples were analyzed to validate the reliability of the microfluidic channel in a clinical application. Wu et al. developed a paper-based microfluidic immunodevice that employed electrochemical- and fluorescent-mediated signal amplification for CTC detection (Figure 6b) [73]. Under optimal conditions, the detection limit of this novel immunodevice was as low as 10 cells/mL.

On the other hand, the rarity of CTCs and CCSCs is the leading limiting factor for clinical commercialization. To overcome this issue, additional detection of other circulating cancer biomarkers such as exosomes and circulating tumor DNA (ctDNA) can be considered. Exosomes play a critical role in a communication system between cells, carrying several biomolecules from one cell to another [89,90,91]. In cancer conditions, cancer cell-derived exosomes are secreted into the bodily fluids with high stability and in higher amounts than the normal cells [92]. Furthermore, the basic concept of exosome detection is the same as CTC detection. For the development of exosome-based early diagnosis and analysis of cancers, tetraspanins (such as CD9, CD63, CD81, and CD82) are typically utilized as the capturing molecules for the detection of cancer-associated exosomes. For cancer-related antigens on the lipid bilayer of exosomes, many different proteins can be utilized as biomarkers, depending on the host cancer cells, including HER2, CEA, EpCAM, IGFR, PSMA, etc., which are used as diagnostic and therapeutic markers. Therefore, the detection method of exosomes is very similar to that of CTCs and CCSCs, so the same device can be applied for both CTC/CCSCs and exosomes. Consequently, by detecting both circulating cancer cells and exosomes together, the rarity issue will be mitigated.

## 6. Conclusions

In conclusion, we review recent efforts made to integrate nanotechnology-based optical biosensors with microfluidic systems for the detection of CTCs and CCSCs. Each method has unique properties and optimal target conditions for successful detection. For this purpose, isolation, enrichment, capture, and post-sensing steps should take into consideration the type of CTCs or CCSCs to be detected. With CTC/CCSC enumeration, it can be directly applied to assist in identifying early cancer treatment response and prognosis [70,95]. In particular, a recent study with human patients showed that the number of CCSCs is more critical than CTCs in the overall survival periods, respectively [95]. Since the differentiation condition of CSC in vivo and in vitro is difficult to exactly match, it is limited in predicting the exact cancer phenotype of differentiated CSCs. However, we found that differentiated CSCs’ surface marker profiles were similar to the tissue samples of the secondary tumor in vivo [70]. Regarding this result, we believe that the detection and analysis of CCSCs has great potential to contribute to clinical applications for cancer treatment.

## Figures and Tables

**Table 1:** Comparison of various strategies for the enrichment of CTCs with their specific structure and enrichment efficiency.

Enrichment Strategy	Developed Structures	Target	Sample Type	Enrichment Efficiency	Hands-On-Time	Advantages	Limitations	Clinical Trial	Ref.
Nanoparticle-assisted enrichment strategy	Wavy-herringbone (HB)-structured microfluidic device with anti-epithelial cell adhesion molecule (EpCAM)-coated magnetic particles.	CTCs	Whole blood	81–95%	Within 1 h	High isolation efficiency	Wide variation in isolation efficiency	No	[40]
	Volumetric bar-chart chip (V-Chip) with magnetic bead-labeled aptamer-conjugated nanoparticles (ACNPs)	CTCs	In buffer with human white blood cells (WBCs)	Single-cell detectable	20 min	Portable, quantitative detection with WBCs (high background)	Need several steps for isolation	No	[41]
Direct capturing on the nano- and microstructures	Surface area increased 3D-printed microfluidic device functionalized with anti-EpCAM antibodies	CTCs	Spiked human blood sample	Capture efficiency of 95% in spiked human blood samples	Within 1 h (Optimal flow rate: 1 mL/h)	High isolation efficiency	Over 25% of EpCAM-negative CTC isolation	No (Healthy donor blood)	[45]
Density-based isolation	Microfluidic chip of size-based separation, capture, staining, or in situ culture of cells	CTCs	Spiked human blood sample	Capture efficiency of 70% in the blood sample	8 min	Label-free and rapid isolation	Low isolation efficiency, low survival ratio after isolation	No (Healthy donor blood)	[50]

**Table 2:** Common cell surface markers to identify CTCs and CCSCs.

Origin of Cancer	CTC Markers	Ref.	CCSC Markers	Ref.
In general	EpCAM^+^ or Cytokeratin^+^, CD45^−^	[54]		
Brain cancer (glioblastoma)	EGFR^+^, Ki67^+^ or EB1^+^ MCAM^+^ or MCSP^+^	[62] [63]	SSEA1^+^, CD133^+^	[55]
Breast cancer	EpCAM^+^, HER2^+^, EGFR^+^	[64]	CD44^+^/CD24^low/^^−^, CD133^+^	[70]
Lung cancer	Folate receptor^+^	[65]	CD133^+^	[60]
Liver cancer	ASGPR, GPC3, and EpCAM	[66]	CD133^+^/CD44^+^, CD90^+^	[68]
Gastric cancer	HER2^+^	[69]	CD133^+^/CD44^+^	[59]
Colorectal cancer	CK20^+^, CEA^+^	[67]	CD133^+^/CD44^+^/ESA^high^, CD166^+^, CD26^+^	[58]
Pancreatic cancer	CA19-9^+^	[71]	CD44^+^/CD24^+^/ESA^+^, CD133^+^	[57]
Prostate cancer	PSMA^+^	[72]	CD44^+^	[56]
Ovarian cancer	CA124^+^, HE4^+^	[73]	ALDH1^+^/CD44^+^ or CD133^+^	[61]
